# Immunotherapeutic advances in glioma management: The rise of vaccine‐based approaches

**DOI:** 10.1111/cns.70013

**Published:** 2024-08-30

**Authors:** Wireko Andrew Awuah, Muhammad Hamza Shah, Joecelyn Kirani Tan, Sruthi Ranganathan, Vivek Sanker, Kwadwo Darko, Pearl Ohenewaa Tenkorang, Bryan Badayelba Adageba, Arjun Ahluwalia, Vallabh Shet, Nicholas Aderinto, Mrinmoy Kundu, Toufik Abdul‐Rahman, Oday Atallah

**Affiliations:** ^1^ Faculty of Medicine Sumy State University Sumy Ukraine; ^2^ School of Medicine Queen's University Belfast Belfast UK; ^3^ Faculty of Medicine University of St Andrews St. Andrews UK; ^4^ Department of Medicine University of Cambridge Cambridge UK; ^5^ Department of Neurosurgery Trivandrum Medical College Trivandrum Kerala India; ^6^ Department of Neurosurgery Korle Bu Teaching Hospital Accra Ghana; ^7^ University of Ghana Medical School Accra Ghana; ^8^ Kwame Nkrumah University of Science and Technology School of Medicine and Dentistry Kumasi Ghana; ^9^ Faculty of Medicine Bangalore Medical College and Research Institute Bangalore Karnataka India; ^10^ Department of Internal Medicine LAUTECH Teaching Hospital Ogbomoso Nigeria; ^11^ Institute of Medical Sciences and SUM Hospital Bhubaneswar Odisha India; ^12^ Department of Neurosurgery, Hannover Medical School Hannover Germany

**Keywords:** glioma, glioma vaccines, immunotherapy, neuro‐oncology

## Abstract

**Background:**

Gliomas, particularly glioblastoma multiforme (GBM), are highly aggressive brain tumors that present significant challenges in oncology due to their rapid progression and resistance to conventional therapies. Despite advancements in treatment, the prognosis for patients with GBM remains poor, necessitating the exploration of novel therapeutic approaches. One such emerging strategy is the development of glioma vaccines, which aim to stimulate the immune system to target and destroy tumor cells.

**Aims:**

This review aims to provide a comprehensive evaluation of the current landscape of glioma vaccine development, analyzing the types of vaccines under investigation, the outcomes of clinical trials, and the challenges and opportunities associated with their implementation. The goal is to highlight the potential of glioma vaccines in advancing more effective and personalized treatments for glioma patients.

**Materials and Methods:**

This narrative review systematically assessed the role of glioma vaccines by including full‐text articles published between 2000 and 2024 in English. Databases such as PubMed/MEDLINE, EMBASE, the Cochrane Library, and Scopus were searched using key terms like “glioma,” “brain tumor,” “glioblastoma,” “vaccine,” and “immunotherapy.” The review incorporated both pre‐clinical and clinical studies, including descriptive studies, animal‐model studies, cohort studies, and observational studies. Exclusion criteria were applied to omit abstracts, case reports, posters, and non‐peer‐reviewed studies, ensuring the inclusion of high‐quality evidence.

**Results:**

Clinical trials investigating various glioma vaccines, including peptide‐based, DNA/RNA‐based, whole‐cell, and dendritic‐cell vaccines, have shown promising results. These vaccines demonstrated potential in extending survival rates and managing adverse events in glioma patients. However, significant challenges remain, such as therapeutic resistance due to tumor heterogeneity and immune evasion mechanisms. Moreover, the lack of standardized guidelines for evaluating vaccine responses and issues related to ethical considerations, regulatory hurdles, and vaccine acceptance among patients further complicate the implementation of glioma vaccines.

**Discussion:**

Addressing the challenges associated with glioma vaccines involves exploring combination therapies, targeted approaches, and personalized medicine. Combining vaccines with traditional therapies like radiotherapy or chemotherapy may enhance efficacy by boosting the immune system’s ability to fight tumor cells. Personalized vaccines tailored to individual patient profiles present an opportunity for improved outcomes. Furthermore, global collaboration and equitable distribution are critical for ensuring access to glioma vaccines, especially in low‐ and middle‐income countries with limited healthcare resources

**Conclusion:**

Glioma vaccines represent a promising avenue in the fight against gliomas, offering hope for improving patient outcomes in a disease that is notoriously difficult to treat. Despite the challenges, continued research and the development of innovative strategies, including combination therapies and personalized approaches, are essential for overcoming current barriers and transforming the treatment landscape for glioma patients.

## Graphical abstract

The complexity of gliomas, including challenges like blood–brain barrier (BBB) breakdown, tumor heterogeneity, and immune evasion, can be addressed through the development of glioma vaccines. These vaccines aim to enhance the immune system's ability to recognize and target tumor‐associated antigens, leading to a more effective antitumor response. The various vaccine strategies—dendritic cell‐based, peptide‐based, and viral vector‐based—are discussed, along with their potential in overcoming the limitations of traditional therapies.

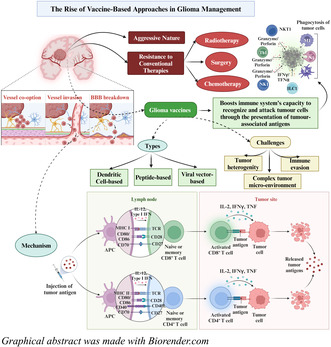

## 1 INTRODUCTION

The central nervous system (CNS), comprising neurons and glial cells, is crucial for maintaining neurological homeostasis. Glial cells, when malignantly transformed, give rise to gliomas, the most common primary brain tumors, characterized by their glial origin—astrocytes, oligodendrocytes, and ependymal cells. Glioblastoma multiforme (GBM), the most aggressive form of gliomas, presents a formidable challenge in treatment due to its heterogeneity, invasive growth, and the CNS's protective microenvironment, which shields these tumors from conventional therapies and immune attacks.^1^, ^2^

Traditional clinical management of gliomas involves surgical resection, complemented by radiotherapy, and chemotherapy. However, the prognosis remains bleak, attributed to the tumor's resistance, the blood–brain barrier (BBB) impeding therapeutic delivery, and an immunosuppressive microenvironment, all of which contribute to recurrent resistance to treatment.^3^, ^4^ Amidst these challenges, glioma vaccines have emerged as a promising therapeutic avenue, leveraging the immune system to target and eliminate tumor cells. By introducing tumor‐associated antigens to the immune system, these vaccines aim to elicit a targeted immune response against the tumor. The exploration of peptide‐based, dendritic cell‐based, and viral vector‐based vaccines has shown promise in enhancing immune recognition of glioma cells and generating specific, lasting immune responses, marking a pivotal shift toward immunotherapy in the glioma treatment paradigm.^5^, ^6^

Through a critical analysis of recent progress and existing obstacles, this review aims to illuminate the potential of glioma vaccines, reviewing the current landscape of vaccine development, examining the scientific underpinnings, outcomes from clinical trials, and the vaccines' emerging role in glioma therapy in advancing more effective and personalized treatment approaches for patients with gliomas.

## 2 METHODOLOGY

This narrative review seeks to comprehensively assess the role of glioma vaccines, employing specific inclusion and exclusion criteria to ensure a thorough analysis. Inclusion criteria encompassed full‐text articles in English published between 2000 and 2024, chosen to allow a comprehensive evaluation of established practices and capture significant advancements over an extended period. Multiple databases, including PubMed/MEDLINE, EMBASE, the Cochrane Library, and Scopus, underwent systematic searches to establish a comprehensive literature base.

Utilizing key search terms such as “glioma,” “brain tumor,” and “glioblastoma” in conjunction with specific terms like “vaccine” and “immunotherapy” ensured the inclusion of pertinent articles. In addition to the systematic database search, a manual examination of references cited in recent glioma vaccine reviews identified supplementary sources. Exclusion criteria were applied to exclude standalone abstracts, case reports, posters, and unpublished or non‐peer‐reviewed studies, prioritizing high‐quality, reliable evidence.

The review's scope did not impose restrictions on the number of included studies, aiming for a comprehensive understanding and encompassing diverse study designs. The review integrates descriptive studies, animal‐model studies, cohort studies, and observational studies, providing a holistic perspective on the application of glioma vaccines. Both pre‐clinical and clinical studies were incorporated to broaden the scope of knowledge covered in this review. A summary of the review's methodology is depicted in Table 1.

**TABLE 1:** Summary of methodology for this review.

Methodology steps	Description
Literature Search	PubMed/MEDLINE, EMBASE, the Cochrane Library, and Scopus
Inclusion Criteria	Full‐text articles published in English Publication date range: 2000–2024 Focus on glioma vaccines
Exclusion Criteria	Standalone abstracts Case reports Posters Unpublished or non‐peer‐reviewed studies
Search Terms	Key search terms such as “glioma,” “brain tumor” and “glioblastoma” were used alongside specific terms like “vaccine,” and “immunotherapy”
Additional Search	Manual examination of references cited in recent disease‐specific reviews No predetermined limit on the number of studies Encompassing diverse study designs: Descriptive studies Animal‐model studies Cohort studies Observational studies Including investigations in both pre‐clinical and clinical settings

## 3 BACKGROUND ON GLIOMAS

### 3.1 Glioma classifications

The World Health Organization's (WHO's) CNS5 classification system represents a pivotal evolution in the classification of gliomas by distinguishing between “adult‐type” and “pediatric‐type” gliomas. This significant development stems from years of clinical observation and advancements in molecular research, which have elucidated the distinct molecular landscapes characteristic of gliomas in adults and children, thereby enabling a more precise categorization based on both clinical behavior and biological characteristics.^7^, ^8^

Incorporating both histopathological and molecular criteria, the WHO's CNS5 classification organizes brain tumors into six distinct families based on unique features and behaviors. Adult‐type diffuse gliomas, including GBM and IDH‐wildtype tumors, are noted for their prevalence and the challenges they present in adult neuro‐oncology.^7^, ^9^ Conversely, pediatric‐type diffuse low‐grade gliomas (LGGs) are generally associated with a more favorable prognosis, highlighting the importance of molecular diagnostics in distinguishing between tumor types to inform treatment strategies.^10^ Pediatric‐type diffuse high‐grade gliomas (HGGs), known for their aggressive behavior and poorer outcomes, underscore the heterogeneity within pediatric gliomas and the critical need for targeted therapeutic approaches.^11^ Furthermore, this classification also acknowledges circumscribed astrocytic gliomas, characterized by their more defined growth patterns compared to the inherently diffuse nature of tumors in other families. This differentiation aids in distinguishing them from more invasive gliomas, influencing surgical, and therapeutic decision‐making.^7^, ^12^

Overall, the integration of molecular diagnostics in the WHO CNS5 system's glioma classification not only enriches our understanding of tumor biology but also significantly impacts clinical practice. By differentiating between adult and pediatric gliomas, it allows for treatment strategies tailored to the specific molecular profile of each tumor, potentially improving patient outcomes. This approach underscores the importance of precise molecular diagnostics in prognostication and in the development of targeted therapies, marking a shift toward more personalized medicine in the management of gliomas.

### 3.2 Current treatment modalities for gliomas

#### 3.2.1 Conventional treatment

Current treatments for gliomas, especially GBM, blend conventional methods with innovative strategies to enhance patient outcomes. These tumors challenge treatment due to their aggressiveness, intricate tumor microenvironment, and the BBB, which hampers drug delivery.

Standard care involves surgical resection, radiation therapy, and chemotherapy, primarily using temozolomide (TMZ). Despite efforts, improvements in survival are modest, with GBM's high recurrence rates linked to glioblastoma stem cells (GSCs) and the tumor's invasive nature, complicating complete removal. Advances in intraoperative imaging have improved tumor margin definition, yet complete resection remains elusive.^13^, ^14^

Post‐surgical management of LGG may adopt a “watch‐and‐wait” strategy for younger patients without seizure history. For older patients or those with residual tumors, radiotherapy is recommended, improving seizure control and progression‐free survival (PFS) without affecting overall survival. TMZ chemotherapy is an alternative when radiotherapy is unsuitable, although its PFS benefits are less pronounced for certain low‐grade astrocytomas.^15^, ^16^

For higher‐grade astrocytomas (WHO grade 3–4), a 60 Gy radiotherapy dose is advised. The EORTC 26053 (CANTON) trial indicated no benefit from concurrent TMZ but showed improved survival with adjuvant TMZ in IDH‐mutant glioma cases, necessitating further investigation into TMZ's optimal use.^1^, ^17^ Additionally, adding PCV chemotherapy to radiotherapy has been shown to improve OS in patients with oligodendroglioma, 1p/19q‐codeleted.^18^ Two major randomized controlled trials, EORTC 26951 and RTOG 9402, demonstrated a significant survival benefit of 5–6 years when PCV was included in the treatment regimen.^18^, ^19^ However, alkylating chemotherapy alone, such as TMZ or PCV, did not produce similar outcomes compared to radiotherapy combined with PCV. The standard treatment approach for this patient group is PCV followed by radiotherapy, although compliance with completing the full PCV treatment cycles has been challenging.^20^ The use of TMZ in combination with radiotherapy is being investigated as a potential alternative to PCV in the modified CODEL trial, with hopes of achieving similar or better outcomes.^21^

#### 3.2.2 Novel approaches

Immunotherapy has emerged as a promising treatment modality for gliomas, leveraging the body's immune system to recognize and attack tumor cells. Despite the immunosuppressive environment of gliomas, various strategies are being explored, including the use of immune checkpoint inhibitors, vaccination, and adoptive cell therapy. The combination of immunotherapy with conventional treatments is being actively investigated to overcome the limitations of each approach and achieve synergistic effects.^22^, ^23^ Clinical trials are currently underway to explore the potential of immunotherapy in GBM treatment, although challenges such as tumor heterogeneity and immune suppression by the tumor still need to be addressed.^24^

Stem cell‐based therapy also offers a novel approach to specifically targeting tumor cells while sparing healthy brain tissue. Stem cells can be engineered to deliver therapeutic agents directly to the tumor site, potentially establishing a long‐term antitumor response. This strategy is still in the experimental stages but holds promise for providing targeted and effective treatment for gliomas.^25^ Additionally, advancements in understanding the molecular mechanisms underlying glioma progression have led to the development of targeted therapies aimed at specific genetic and molecular aberrations within the tumor. These include targeting tyrosine kinase receptors, the PI3K/AKT pathway, and other critical signaling pathways involved in glioma pathogenesis. Novel approaches such as CRISPR/Cas9 for gene editing and RNA interference are also being explored to silence oncogenes and activate tumor suppressor genes.^26^

Not only this, but nanoparticles have also been investigated for their ability to cross the BBB and deliver therapeutic agents directly to glioma cells. Hyperthermia therapy using magnetic nanoparticles, gold nanorods, or carbon nanotubes aims to induce tumor cell death by raising the temperature at the tumor site. This approach can be combined with radiation therapy or chemotherapy to enhance treatment efficacy.^27^

### 3.3 Limitations to effective glioma treatment

The treatment of HGGs is complicated by their infiltrative nature, making it challenging to delineate precise anatomical borders during neurosurgical resections, a difficulty exacerbated by the lack of definitive markers for distinguishing tumors from normal brain tissue at the histological level.^28^, ^29^ Innovations such as intraoperative MRI and fluorescence imaging are under investigation to improve visualization of tumor extent during surgery.^30^ In addition, the BBB presents another significant obstacle, with its integrity variably affected by glioma progression, complicating drug delivery to tumor sites.^31^, ^32^, ^33^, ^34^ Many therapeutic agents demonstrate an inability to cross the BBB effectively, limiting their utility in treatment.^35^

In the same token, gliomas' heterogeneity further complicates therapeutic development, with variability in cell types, mutations, and adaptation to therapeutic stress, leading to dynamic changes in the tumor's cellular and mutation landscape.^36^, ^37^, ^38^, ^39^, ^40^, ^41^ Similarly, resistance to therapies, whether intrinsic or acquired, poses a substantial challenge in glioma treatment. Both forms of resistance involve common molecular pathways, including drug efflux mechanisms and dysregulation of miRNAs, complicating the development of effective treatments.^42^, ^43^ Moreover, the absence of reliable biomarkers for early glioma detection hampers diagnosis and management, emphasizing the need for biomarkers that can be detected in liquid biopsies for routine clinical use.^44^

Finally, the immunosuppressive tumor microenvironment (TME) in gliomas, characterized by altered immune cell behavior and T‐cell dysfunction, significantly undermines immunotherapeutic strategies and tumor elimination efforts.^45^, ^46^, ^47^

## 4 RATIONALE FOR DEVELOPING GLIOMA VACCINES

Vaccine therapies represent a groundbreaking shift in glioma treatment, overcoming the limitations of current modalities through their specificity, immunological memory, minimal toxicity, combinatory potential, personalized approach, and prophylactic capabilities. These vaccines offer a refined treatment strategy, minimizing harm to healthy cells while optimizing the immune system's attack on tumor cells. Their compatibility with existing therapies and potential for preventive application herald a transformative approach in cancer care.

The SurVaxM peptide vaccine, targeting survivin prevalent in GBM cells, exemplifies this specificity.^48^ For IDH1(R132H) + astrocytomas, vaccines targeting the IDH1(R132H) mutation advance precision medicine.^49^ Vaccines like APVAC1 and personalized neoantigen‐targeting formulations have been shown to induce durable immune memory, enabling rapid immune responses to tumor recurrences, and thus reducing relapse risks.^50^, ^51^

**FIGURE 1:**
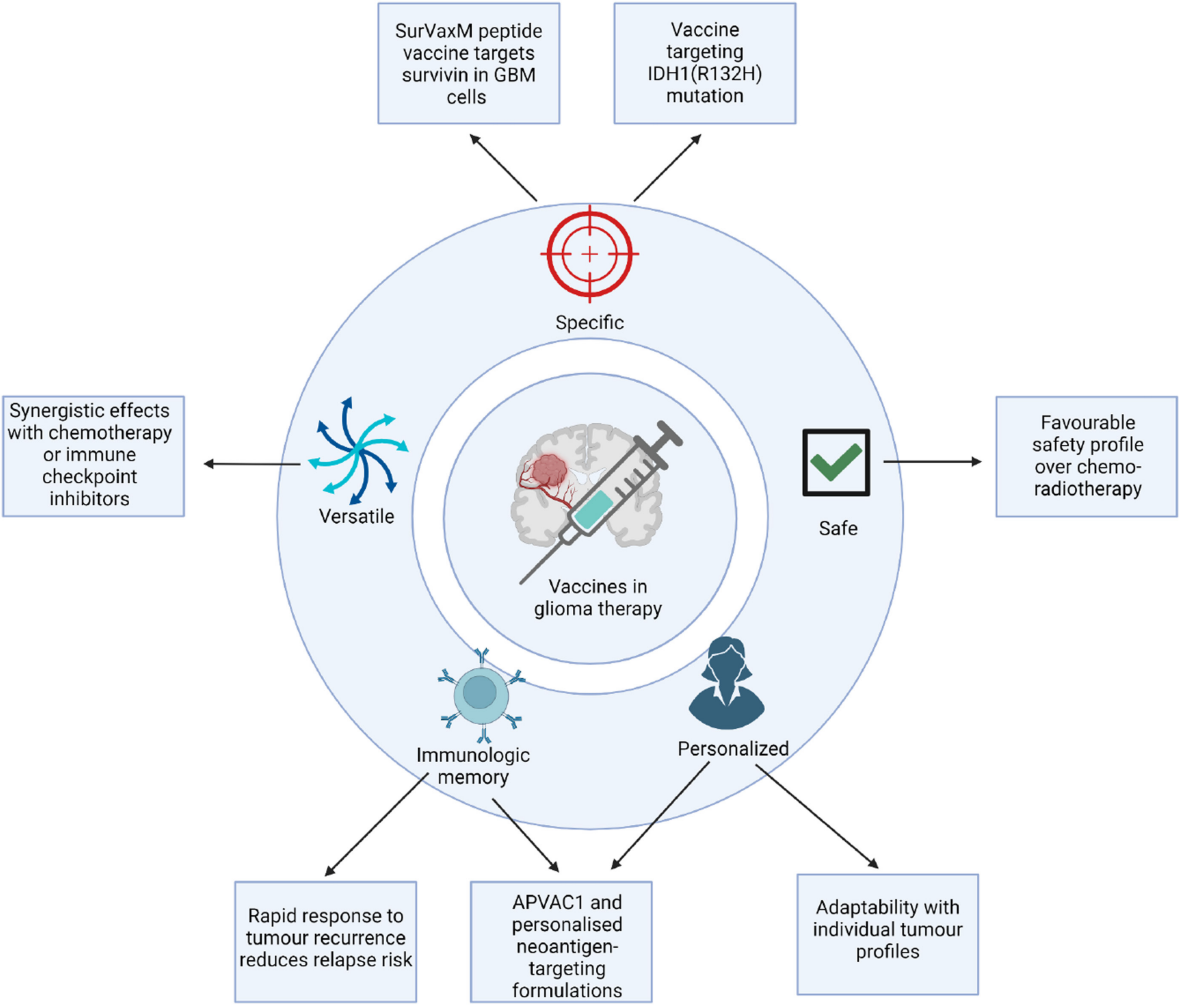
Comprehensive framework for the rationale behind glioma vaccine development. Image was created with Biorender.com. APVAC, Actively personalized vaccines; GBM, Glioblastoma Multiforme; IDH, Isocitrate Dehydrogenase.

Contrasting with the systemic side effects common in chemotherapy and radiation, vaccine therapy generally presents a favorable safety profile.^52^, ^53^, ^54^ The potential for synergistic effects with other treatments, such as chemotherapy or immune checkpoint inhibitors, further underscores vaccines' versatility.^55^ Additionally, the adaptability of vaccine therapy to individual tumor profiles offers a bespoke treatment strategy, recognizing each patient's unique tumor biology. The proactive potential of vaccines, informed by genetic sequencing to identify and target mutations before glioma development, suggests a role in preventing gliomas in high‐risk individuals, marking a proactive stance in cancer management. Figure 1 showcases the aforementioned points in a diagrammatic format.

## 5 GLIOMA VACCINES

### 5.1 Glioma immunology and pathophysiological basis of vaccines

Histopathological and flow cytometry analyses of gliomas in human and rodent models have disclosed a complex microenvironment teeming with various cell types, including reactive astrocytes, endothelial cells, and a spectrum of immune cells. This immune landscape encompasses microglia, peripheral macrophages, granulocytes, myeloid‐derived suppressor cells (MDSCs), and T lymphocytes, with glioma‐associated microglia and macrophages (GAMs) and MDSCs predominating in aggressive gliomas. Their abundance correlates inversely with patient survival, underscoring a significant impact on prognosis.^56^, ^57^

GAMs exhibit compromised immune functionality, lacking in innate immune triggers, cytokine production, and co‐stimulatory molecule expression. These, alongside MDSCs, contribute to an immunosuppressive milieu by secreting cytokines and chemokines that modulate antitumor responses. Furthermore, these cells facilitate the recruitment of regulatory T cells to the tumor site, with MDSCs specifically impairing the activity of natural killer cells and the activation of tumor‐reactive T cells.^58^, ^59^, ^60^

The intricate immune evasion tactics of gliomas present substantial therapeutic hurdles, emphasizing the critical need for a deeper understanding of the immune components involved. Glioma cells, along with recruited immune cells, further mediate an environment conducive to immune evasion, complicating effective treatment strategies. While certain immune cells hold the potential for tumor suppression, others, particularly immunosuppressive populations, bolster gliomas' ability to elude immune detection.^61^, ^62^

**FIGURE 2:**
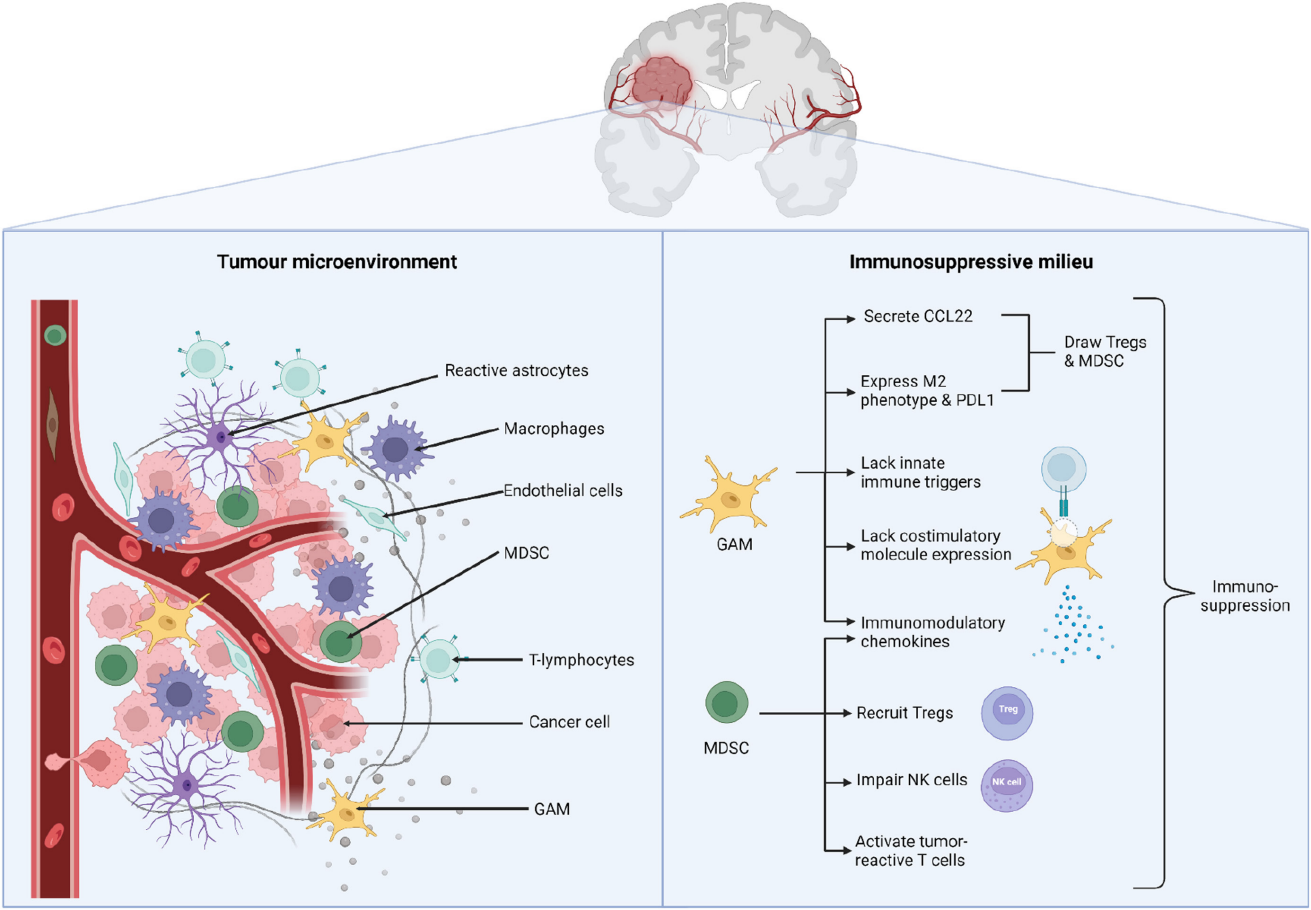
Overview of the tumor microenvironment and immunosuppressive foundations in glioma immunology. Created with Biorender.com. CCL22, CC Motif Chemokine Ligand 22; GAMs, Glioma‐associated microglia and macrophages; MDSC, Myeloid‐derived suppressor cells; PDL1, Programmed death‐ligand 1.

MDSCs, especially the monocytic subtype prevalent in GBM tumors, are instrumental in fostering gliomas' immunosuppressive state. These cells attract CD4+ regulatory T cells, known for their immunosuppressive effects, further dampening immune responses against the tumor. The significant presence of regulatory T cells in glioma patients, both in peripheral blood and within tumors, along with the downregulation of costimulatory molecules, correlates with reduced recurrence‐free survival. Moreover, GAMs, expressing the immunosuppressive M2 phenotype and PD‐L1, secrete CCL22, drawing regulatory T cells, and MDSCs into the tumor microenvironment, thereby enhancing local immunosuppression.^63^, ^64^, ^65^ This intricate interplay between glioma cells and the immune system highlights the complexities of glioma immunobiology and underscores the necessity for innovative therapeutic approaches that can effectively navigate and counteract this challenging landscape. Figure 2 summarizes the aforementioned TME and immunosuppressive basis of glioma immunology.

### 5.2 Insights into glioma vaccine types, structural components, and their mechanisms of action

Glioma vaccines can be classified as peptide‐based vaccines, nucleic acid‐based vaccines, whole‐cell vaccines, and dendritic‐cell vaccines. These subcategories of glioma vaccines differ in their structural components, and therefore their mechanism of action against the tumor cells.

#### 5.2.1 Peptide‐based vaccines

Peptide‐based vaccines in glioma treatment are designed to elicit an immune response specifically targeted at tumor cells by using peptides derived from glioma‐associated antigens.^66^ These peptides are taken up by antigen‐presenting cells (APCs) like dendritic cells, processed, and then presented on their surface in conjunction with major histocompatibility complex (MHC) molecules.^67^ This process is crucial for the subsequent activation of T cells, including both cytotoxic CD8+ T cells, which can directly kill tumor cells presenting the antigen, and helper CD4+ T cells, which support the immune response by producing cytokines that promote the proliferation and activation of CD8+ T cells and B cells.^68^ Adjuvants like poly‐ICLC are often included in peptide vaccines to enhance the immune response. Poly‐ICLC acts as a potent stimulator of type I interferon production, which further activates immune cells and improves the efficacy of the vaccine.^67^

The clinical potential of peptide‐based vaccines in gliomas is further supported by clinical trials. For instance, a study by Migliorini et al.^66^ discusses the safety and immunogenicity of the IMA950 multipeptide vaccine adjuvanted with poly‐ICLC in glioma patients, highlighting the vaccine's ability to induce specific T‐cell responses. Additionally, the work by Horwacik et al.^67^ on peptide mimetics offers insights into the optimization of peptide–antibody interactions, a crucial aspect of vaccine design that can influence immunogenicity and therapeutic outcomes.

#### 5.2.2 DNA/RNA‐based vaccines

DNA and RNA vaccines operate by engaging both innate and adaptive immune mechanisms, offering a novel approach in the fight against gliomas. RNA vaccines, upon administration, leverage host cell machinery to synthesize tumor‐specific antigens without integrating into the host DNA, ensuring both safety and specificity.^69^ The antigens produced are presented on the cell surface via MHC molecules, initiating an immune response. This process is further bolstered by the innate immune system through pathways such as the retinoic acid‐inducible gene I (RIG‐I), enhancing the vaccine's immunogenicity by recognizing viral RNA components.^70^ On the other hand, DNA vaccines have been extensively tested and applied against various pathogens and tumors over the past 2 decades.^71^ They offer a conceptually safe, non‐live vaccine approach that can induce both humoral and cellular immune responses, including the elusive target of killer cytotoxic T lymphocytes (CTLs).^72^ The DNA vaccine approach offers a conceptually safe, technically simple, and promising alternative to traditional live and killed vaccine platforms by overcoming the key safety concerns associated with reversion risks, potential spread, and manufacturing risks.^72^

In gliomas, these vaccines target tumor‐specific antigens or mutations, such as the H3K27M mutation in diffuse midline gliomas (DMGs), to drive a targeted immune response, capitalizing on the tumors' unique genetic alterations.^70^ By conveying the genetic blueprints (mRNA or DNA) for tumor antigens directly into host cells, these vaccines prompt the body to produce these antigens internally, leading to immune recognition and action.

DNA and RNA vaccines are particularly useful in overcoming the immunosuppressive TME in gliomas that pose a significant challenge to treatment efficacy, by eliciting a robust immune response. These vaccines can improve antigen presentation and stimulate T cell proliferation against these antigens. Furthermore, targeting specific immune checkpoints or manipulating immunosuppressive molecule expression via RNA interference may diminish the tumor‐induced immunosuppression, facilitating an intensified immune assault on tumor cells.^22^ The adaptability of DNA and RNA vaccines to encode precise tumor antigens allows for the customisation of vaccine strategies. Through tumor genetic material sequencing, unique mutations can be pinpointed and targeted, fostering a personalized therapeutic approach. This method ensures that the immune response is selective for tumor cells, minimizing damage to healthy tissue and potentially improving treatment outcomes.^50^

Current clinical trials are evaluating the viability, safety, and effectiveness of DNA and RNA vaccines in glioma treatment. Preliminary trials involving personalized vaccines tailored to individual tumor mutations and antigens have shown encouraging results in provoking specific immune responses against glioma cells, signaling a promising direction for future research and therapy.^50^

#### 5.2.3 Whole‐cell vaccines

Whole‐cell vaccines for gliomas utilize either autologous (from the patient) or allogeneic (from a donor or cell line) tumor cells as a source of antigens, aiming to induce a robust immune response against the tumor. These vaccines are designed to present a broad array of glioma‐associated antigens to the immune system, some of which may be unique to the patient's tumor, thereby personalizing the treatment. The composition of whole‐cell vaccines includes the entire repertoire of tumor antigens present in glioma cells.^55^ This comprehensive antigenic profile can stimulate a broad immune response, targeting multiple epitopes on the tumor cells. To prepare the vaccine, tumor cells are collected either from surgical resection of the patient's glioma (autologous) or from established glioma cell lines (allogeneic). These cells are then inactivated, typically by irradiation, to prevent further tumor growth upon re‐administration to the patient. Additional treatments, such as genetic modifications to increase immunogenicity or the addition of adjuvants like granulocyte‐macrophage colony‐stimulating factor (GM‐CSF), may be employed to enhance the vaccine's effectiveness.^55^

The mechanism of action of whole‐cell glioma vaccines is multifaceted. Upon administration, the inactivated tumor cells are taken up by antigen‐presenting cells (APCs), such as dendritic cells. These APCs process the tumor antigens and present them on their surface in the context of major histocompatibility complex (MHC) molecules. The presentation of glioma antigens by APCs activates both helper T cells (CD4+) and cytotoxic T cells (CD8+). Helper T cells facilitate the activation and proliferation of cytotoxic T cells and B cells.^22^ Cytotoxic T cells directly target and kill tumor cells displaying the same antigens. B cells may produce antibodies that bind to tumor antigens, marking them for destruction. Whole‐cell vaccines aim not only to initiate an immediate immune response against gliomas but also to establish immunological memory. This enables the immune system to quickly respond to tumor antigens upon any future encounters, potentially preventing tumor recurrence.^22^ The TME of gliomas is characterized by immunosuppressive mechanisms that protect the tumor from immune detection and destruction. Whole‐cell vaccines, particularly those modified to express immunostimulatory cytokines or to downregulate immunosuppressive factors (e.g., TGF‐β), work to reverse this immunosuppression and render the tumor more susceptible to immune‐mediated attack.^22^

#### 5.2.4 Dendritic‐cell vaccines

Dendritic‐cell (DC) vaccines represent a tailored approach to immunotherapy for gliomas, exploiting the body's immune defenses to target and destroy tumor cells. These vaccines utilize DCs, potent antigen‐presenting cells essential for initiating a robust immune response against malignancies.

The creation of these vaccines involves isolating dendritic cells from the patient's blood through leukapheresis, followed by culturing and maturing these cells in vitro to enhance their antigen‐presenting capabilities. The dendritic cells are then loaded with tumor antigens, which can be derived from the patient's own tumor (autologous) or from synthesized peptides that represent glioma‐associated antigens.^73^ This process prepares the dendritic cells to train the immune system to target glioma cells specifically. Once reintroduced into the patient, these antigen‐loaded dendritic cells travel to lymph nodes, where they present the antigens to T cells, sparking an immune response aimed at the tumor.^73^ The mechanism of action for DC vaccines involves multiple steps culminating in an immune attack on the glioma. After administration, the loaded DCs present tumor antigens to naive T cells in the lymph nodes, activating them into effector cells capable of recognizing and destroying glioma cells presenting the same antigens. This approach aims to induce a targeted immune response that minimizes harm to healthy tissues while potentially establishing immunological memory to prevent future tumor recurrence. Studies have shown that this method can extend survival and elicit tumor‐specific immune responses in glioma patients, highlighting its potential as a part of glioma treatment strategies.^74^, ^75^

**FIGURE 3:**
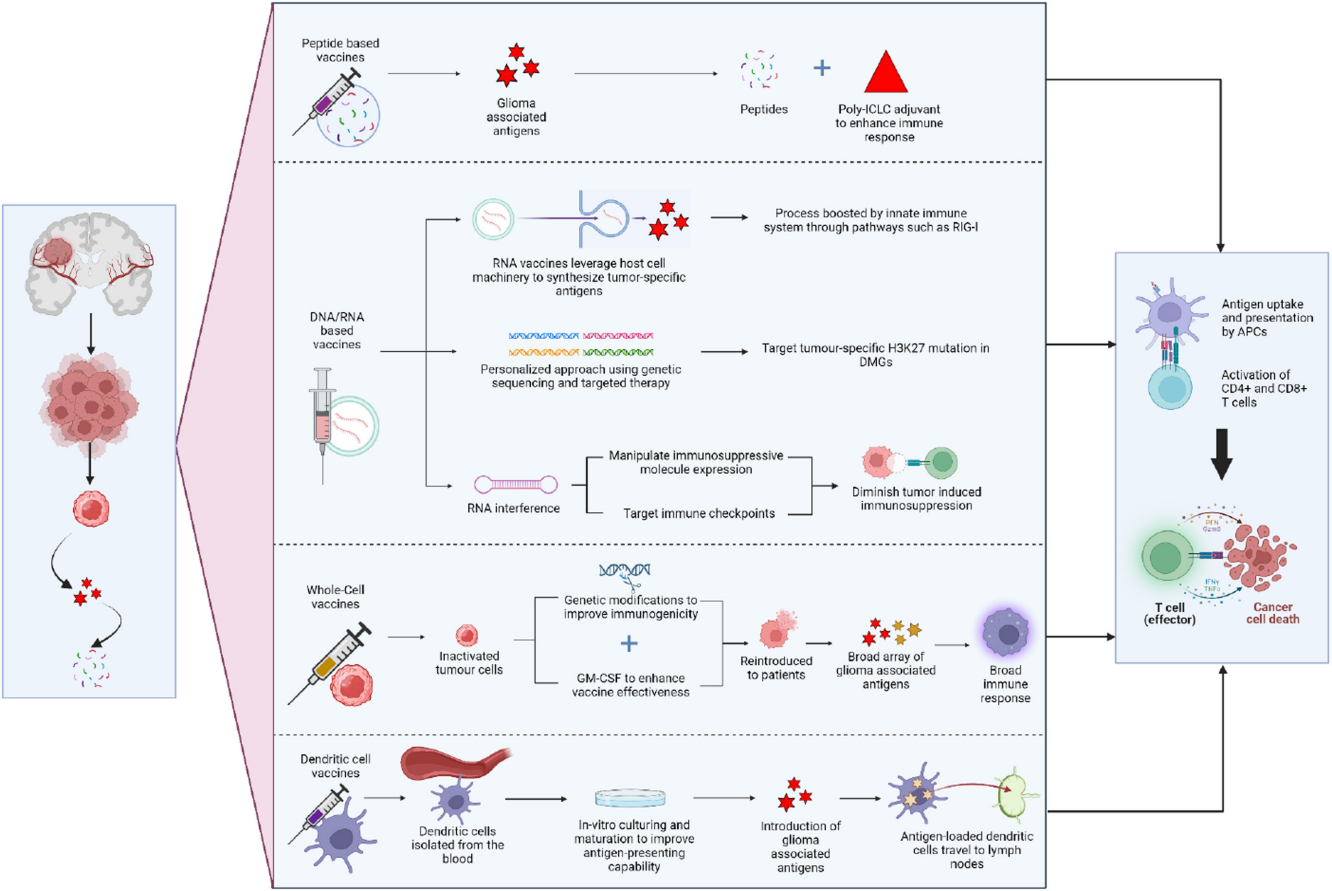
Mechanism of action of glioma vaccines. Image was created with Biorender.com. APCs, Antigen‐Presenting Cells; DMG, Diffuse midline gliomas; Poly‐ICLC, Polyinosinic‐polycytidylic acid; RIG‐1, Retinoic acid‐inducible gene I.

Despite the promise shown by DC vaccines in clinical trials, research continues to optimize the vaccine preparation process, identify the most effective tumor antigens, and understand the best methods for integrating DC vaccines into the broader treatment regimen for gliomas.^75^ The ultimate goal is to improve patient outcomes through a targeted, efficient immune response against glioma cells, making DC vaccines a hopeful avenue for advancing glioma therapy.^75^ Figure 3 illustrates the mechanism of action for the different types of glioma vaccines currently in use.

### 5.3 Similarities and differences among glioma vaccine types

#### 5.3.1 Similarities

##### Immune stimulation

All glioma vaccine approaches aim to activate the patient's immune system to recognize and attack tumor cells. They induce a specific immune response against tumor‐associated antigens.^49^ These vaccines use either the patient's own immune system or donor‐derived immune cells to induce a strong anti‐tumor response.^76^ This begins with the presentation of tumor antigens by the antigen‐presenting cells (such as DCs in the case of DC‐based vaccines) to naive T cells in the lymph nodes that enables the activation of both helper CD4+ T cells and cytotoxic CD8+ T cells. Helper T cells facilitate the activation and proliferation of cytotoxic T cells and B cells.^22^ Cytotoxic T cells directly target and kill tumor cells displaying the same antigens. B cells may produce antibodies that bind to tumor antigens, marking them for destruction. The activated immune cells can then infiltrate the tumor, recognize the target antigens and mount a cytotoxic response to destroy the cancer cells.^77^ Overall, each glioma vaccine stimulates the body's tumor‐specific immune response.

##### Targeting tumor antigens

Vaccines target tumor‐associated antigens that are expressed on the surface of tumor cells. Common antigens include EGFR, ErbB2, ErbB‐3, ErbB‐4, HSP27, HSP72, HSP73/70, HSP90, and IDH1.^78^, ^79^, ^80^ In general, cancer immunotherapy relies on the ability of the immune system to target specific cancer antigens, and this is also the same for glioma vaccines, regardless of type.

##### Enhancement of antigen presentation

These vaccines are designed to enhance antigen presentation to the immune system either by delivering the antigens directly to the immune system or by activating antigen‐presenting cells.^81^ In particular, the vaccines generate de novo cancer antigen‐specific T cells via professional antigen‐presenting cells.^82^ Many of the vaccine platforms use adjuvants or other techniques to improve the immunogenicity and antigen presentation capabilities of the vaccine.^83^ For example, whole‐cell vaccines can be modified to increase the expression of tumor‐associated antigens or co‐stimulatory molecules on the cell surface.^84^ Dendritic‐cell vaccines exploit the natural antigen presentation capabilities of dendritic cells loaded with tumor antigens to maximize antigen presentation to T cells.^85^ Peptide‐based vaccines often use adjuvants or delivery systems to enhance the stability, uptake, and presentation of tumor‐specific peptides.^86^ Similarly, DNA/RNA vaccines can incorporate elements such as promoters or immunostimulatory sequences to enhance antigen expression and presentation.^87^ The common goal of these approaches is to optimize the immune system's exposure to the targeted tumor antigens. By enhancing antigen presentation, vaccines aim to improve the activation and expansion of tumor‐specific T cells and other anti‐cancer immune responses.

##### Favorable safety profiles

Overall, glioma vaccines have demonstrated relatively favorable safety profiles, with minimal severe adverse events reported in clinical trials.^88^ In contrast to more aggressive approaches such as chemotherapy or radiation, the vaccines tend to have a lower incidence of serious adverse events.^89^ The vaccines are designed to stimulate the patient's own immune system rather than directly attack tumor cells, resulting in a generally well‐tolerated treatment. This improved tolerability allows for repeated dosing and the potential for combination with other therapies, further increasing the clinical utility of these vaccine approaches.

#### 5.3.2 Differences

##### Unique mechanisms

Nucleic acid‐based vaccines have unique mechanisms that are separate from the antigen presentation pathway outlined above. This involves the activation of pathways specific for RNA and DNA such as RIG‐I and STING, respectively. Other studies have outlined the overall mechanism and the key points of regulation of the RIG‐I and STING pathways, respectively.^90^, ^91^

##### Antigen source

Firstly, whole‐cell vaccines use the patient's own or donor tumor cells as the source of tumor antigens. DNA/RNA vaccines deliver genetic material encoding tumor‐associated antigens, enabling the patient's own cells to produce and present the target antigens.^55^ In addition, peptide‐based vaccines focus on specific, well‐characterized tumor‐associated peptides or proteins as antigenic targets. The peptides are selected on the basis of their immunogenicity and expression on glioma cells.^92^ Dendritic‐cell vaccines use dendritic cells loaded with tumor‐derived antigens, either from the patient's own tumor or from a standardized tumor cell line. The dendritic cells then present these antigens to T cells.^93^

##### Breadth of antigen presentation

Whole‐cell vaccines can present a wider range of tumor antigens, including unknown or uncharacterized antigens. These vaccines have the broadest antigen coverage because they use the patient's own tumor cells or a combination of tumor cells. This provides a comprehensive representation of the diverse array of tumor‐associated antigens present in the patient's glioma.^94^ In contrast, peptide‐based vaccines focus on a more limited set of well‐characterized tumor antigens.^95^ While this targeted approach can be highly specific, it may miss the presentation of other potentially relevant antigens on glioma cells.^95^ These genetically engineered vaccines have the potential for relatively broad antigen coverage as they can be designed to encode multiple tumor‐associated antigens. However, the specific antigens selected for inclusion in the vaccine may still limit the breadth of presentation. In general, DNA/RNA and peptide‐based vaccines target specific, well‐characterized tumor antigens. Dendritic‐cell vaccines can present a range of antigens depending on the source (e.g., whole tumor cells, peptides, or mRNA).^96^ Dendritic‐cell vaccines fall somewhere in between, as they can be loaded with a wider selection of tumor‐derived antigens, either from the patient's own tumor or from a standardized tumor cell line. This allows for more diverse antigen presentation compared to single peptide vaccines.

##### Complexity of manufacturing

Whole‐cell vaccines and dendritic‐cell vaccines require more complex manufacturing processes because they involve the extraction and processing of patient‐specific cells.^97^ This is because the process requires the collection, verification, and processing of patient‐specific tumor samples, which can be logistically challenging and resource intensive. On the other hand, DNA/RNA and peptide‐based vaccines have a relatively simpler manufacturing process.^98^ These genetically engineered vaccines generally have the lowest manufacturing complexity of the glioma vaccine approaches. Production of these vaccines typically involves the synthesis and formulation of DNA or RNA constructs encoding the selected tumor antigens, which can be more easily scaled up and automated. Similarly, peptide‐based vaccines are relatively less complex to manufacture. The production of these vaccines involves the synthesis of specific tumor‐associated peptides, which can be more standardized and scalable compared to the handling of patient‐derived tumor material.

##### Route of administration

Whole‐cell and dendritic‐cell vaccines are typically administered by injection, often intradermally or subcutaneously.^99^ Whole‐cell vaccines are typically administered by intradermal or subcutaneous injection. This route allows the vaccine to be presented directly to the patient's immune system, as the tumor cells can interact with and activate antigen‐presenting cells in the skin or subcutaneous tissue. Dendritic‐cell vaccines are often administered by intradermal or intranodal (direct injection into a lymph node) routes.^100^ These routes are specifically chosen to target dendritic cells, which are key antigen‐presenting cells found in the skin and lymphoid tissues.^100^ DNA/RNA and peptide‐based vaccines can be administered by various routes, including intramuscular, subcutaneous, or intradermal injections.^101^ The choice of route of administration can have a significant impact on the ability of the vaccine to effectively target and activate the desired immune responses. Different routes may preferentially target different immune cell populations and provide different levels of access to the tumor microenvironment. Table 2 summarizes the similarities and differences among glioma vaccine types.

**TABLE 2:** Similarities and differences among glioma vaccine types.

	Peptide‐based vaccine	Nucleic acid‐based vaccine	Whole cell‐based vaccine	DC‐based vaccine
Immune stimulation^49^	All glioma vaccine approaches aim to activate the patient's immune system to recognize and attack tumor cells. They induce a specific immune response against tumor‐associated antigens			
Tumor Antigens^78^, ^79^, ^80^	Involve a targeted response against a specific tumor‐associated protein target			
Safety Profiles^88^, ^89^	Reduced adverse effects in comparison to traditional cancer therapeutic agent			
Unique Mechanisms of Action^90^, ^91^	N/A	Involves the RIG‐I and/or STING pathway	N/A	N/A
Antigen Breadth^55^, ^92^, ^94^, ^96^	Specific antigens	Specific antigens	Wide antigen repertoire	Specific antigens
Complexity of Manufacturing^97^, ^98^	Less complex manufacturing	Less complex manufacturing	More complex manufacturing	More complex manufacturing
Route of Administration^99^, ^100^, ^101^	Subcutaneous injections	Intramuscular, subcutaneous or intradermal injections	Intradermal or subcutaneous injection	Intradermal or subcutaneous injection

## 6 GLIOMA VACCINE APPLICATIONS: OUTCOMES OF CLINICAL TRIALS

### 6.1 Positive outcomes

Clinical trials play a crucial role in evaluating the safety and effectiveness of vaccines in managing gliomas. Research endeavors focusing on vaccines for gliomas, encompassing conditions like GBM, astrocytomas, and diffuse gliomas, have yielded encouraging results. These outcomes include favorable survival rates, prolonged overall survival durations, and a restricted incidence of adverse events.

#### 6.1.1 Peptide‐based vaccines

Peptide‐based vaccines, composed of peptides that elicit immune responses against tumor‐associated antigens, have shown promising outcomes in clinical trials for gliomas.^54^ For instance, the IMA950 vaccine, comprising 11 tumor‐associated peptides (TUMAPs) presented on HLA surface receptors, demonstrated robust activation of the immune system in patients with newly diagnosed HLA‐A*02‐positive GBM. Administered alongside granulocyte macrophage colony‐stimulating factor (GM‐CSF) over a 24‐week period, this vaccine exhibited progression‐free survival rates of 74% at 6 months and 31% at 9 months.^102^ The SurVaxM peptide vaccine conjugate, designed to target survivin, a molecule highly expressed in GBM cells, has shown significant efficacy when administered to patients newly diagnosed with GBM in combination with temozolomide.^48^ The SurVaxM vaccine led to 95.2% of patients remaining progression‐free 6 months after diagnosis. Additionally, the average progression‐free survival was reported to be 11.4 months, with an OS of 25.9 months following the initial dose of SurVaxM.^48^ These findings suggest the potential for SurVaxM to revolutionize the management of GBM, offering a promising avenue for glioma treatment with demonstrated safety and tolerability.

Moreover, the IDH1(R132H)‐specific peptide vaccine (IDH1‐vac) elicited immune responses in 93.3% of patients diagnosed with WHO grade 3 and 4 IDH1(R132H) + astrocytomas, spanning multiple alleles.^49^ The vaccine also demonstrated favorable three‐year progression‐free and death‐free rates of 0.63 and 0.84, respectively. Notably, the vaccine met its primary safety endpoint, with vaccine‐related adverse events limited to grade 1, underscoring its safety and efficacy in treating patients with advanced astrocytomas. Peptide‐based vaccines have demonstrated efficacy in treating DMGs, targeting the H3.3K27M mutation, a shared neoantigen present in HLA‐A*02.01+ and H3.3K27M+ DMGs, including diffuse intrinsic pontine glioma (DIPG).^54^ Administration of the H3.3K27M‐specific vaccine was well tolerated, yielding an OS rate of 40% for DIPG patients and 39% for nonpontine DMG patients at 12 months.^54^ Notably, patients exhibiting H3.3K27M‐specific CD8+ immunological responses demonstrated prolonged OS compared to nonresponders, indicating the efficacy of the H3.3K27M‐specific vaccine in improving the clinical outcome of DMG patients.

#### 6.1.2 DNA/RNA‐based vaccines

DNA and RNA‐based vaccines employ genetic material from the pathogen to stimulate an immune response and confer immunity against infectious diseases or cancer. Investigated by the Glioma Actively Personalized Vaccine Consortium (GAPVAC), these vaccines integrate highly individualized vaccinations targeting both unmutated antigens (APVAC1) and neoepitopes (APVAC2) into standard care for patients with newly diagnosed GBM.^50^ The treatment demonstrated feasibility in patients with newly diagnosed GBM, with unmutated APVAC1 antigens eliciting sustained responses of central memory CD8+ T cells and APVAC2 inducing predominantly CD4+ T cell responses of T helper 1 type against predicted neoepitopes.^50^ This approach yielded enhanced immunogenicity and the generation of memory T cells in patients, paving the way for personalized treatment strategies for GBM patients.

Similarly, another personalized neoantigen‐targeting vaccine has demonstrated the ability to target tumor‐specific mutations and potentially modulate the immune environment to promote tumor rejection.^51^ Consistent with the combined effect observed with APVAC1 and APVAC2, this vaccine elicited the generation of circulating polyfunctional neoantigen‐specific CD4+ and CD8+ T cell responses, enriched in a memory phenotype, and increased infiltration of T cells into the tumor. Furthermore, there is evidence suggesting that neoantigen‐specific T cells from the peripheral blood can migrate into intracranial GBM tumors, indicating the vaccine's potential efficacy in modifying the immune environment of GBM.^51^

#### 6.1.3 Whole‐cell vaccines

Whole‐cell vaccines are composed of whole tumor cells, typically obtained from the patient's own body (autologous), which undergo modifications or irradiation to stimulate an immune response against a wide array of tumor‐associated antigens. In the case of grade IV astrocytoma, a highly malignant brain tumor, a whole‐cell vaccine approach involves genetically altering autologous tumor cells to hinder the secretion of TGF‐beta2, with the intention of impeding mechanisms by which tumors evade the immune system and fostering clinically effective anti‐tumor immune responses.^53^ This treatment demonstrated good tolerability among patients and resulted in the inhibition of TGF‐beta2 secretion by as much as 98%. Moreover, it led to partial regressions in 33% of patients, stable disease in another 33%, overall median survival of 68 weeks, and a median survival of 78 weeks in those patients who responded positively, surpassing the historical survival rates of 47 weeks observed in conventionally treated glioma patients.^53^ The vaccine's capacity to mitigate immunosuppression, trigger anti‐tumor immune responses, yield favorable clinical outcomes, and uphold safety and tolerability highlights its potential to enhance patient prognoses in the management of grade IV astrocytoma.

Moreover, whole‐cell vaccines hold considerable promise for managing recurrent malignant gliomas in patients. Specifically, utilizing irradiated autologous whole tumor cells in conjunction with granulocyte‐macrophage colony‐stimulating factor as an adjuvant has demonstrated efficacy in eliciting cell‐mediated immune responses.^55^ Notably, 89% of patients exhibited a delayed‐type hypersensitivity response to the vaccination regimen, accompanied by radiological evidence of response in 42% of cases and clinical improvement in 26% of patients, with a median survival of 12 months.^55^ Importantly, both the presence of a delayed‐type hypersensitivity response and radiological evidence of response were associated with enhanced survival outcomes, suggesting that the vaccine elicits tumor‐specific immune responses and may contribute to improved survival rates despite the advanced stage of the disease.

The ERC1671 vaccine, comprising whole, inactivated tumor cells combined with tumor cell lysates obtained from the patient and three GBM donors, yielded favorable outcomes in the treatment of recurrent GBM.^103^ Patients administered ERC1671 alongside bevacizumab exhibited a median OS of 12 months, whereas those receiving placebo plus bevacizumab demonstrated a median OS of 7.5 months.^103^ Furthermore, there was a notable correlation between the maximal count of CD4+ T‐lymphocytes and OS in the ERC1671‐treated group, suggesting a potential immunological mechanism contributing to the observed survival benefit. Importantly, the incorporation of ERC1671 alongside bevacizumab led to a clinically significant enhancement in survival outcomes with minimal additional toxicity.^104^ Moreover, the integration of fractionated radiotherapy (FRT) and TMZ therapy alongside autologous formalin‐fixed tumor vaccine (AFTV) constituted a well‐tolerated therapeutic regimen for individuals diagnosed with newly onset GBM.^104^ Notably, a considerable proportion of patients demonstrated progression‐free survival (PFS) exceeding 24 months, accompanied by favorable median PFS and OS rates, suggesting potential efficacy in the management of newly diagnosed GBM cases. Furthermore, patients exhibiting a robust delayed‐type hypersensitivity (DTH) response to AFTV injections manifested extended PFS durations, indicating a potential correlation between immune response and enhanced clinical outcomes.^104^ These findings underscore the necessity for further exploration of whole‐cell vaccine applications in glioma patients with the aim of improving overall patient prognosis.

#### 6.1.4 Dendritic‐cell vaccines

DC vaccinations have emerged as a promising therapeutic avenue for patients afflicted with GBM. One such vaccine, DCVax‐L, utilizes autologous DCs loaded with tumor lysate to engage the patient's immune system in combating the malignancy, heralding a significant shift toward personalized immunotherapy in the fight against this aggressive cancer.^105^ Newly diagnosed GBM patients treated with DCVax‐L reported a median overall survival of 23.1 months post‐surgery across the intent‐to‐treat population—a noteworthy outcome considering the generally bleak prognosis associated with GBM.^105^ Moreover, the integration of DCVax‐L into the existing treatment paradigm for GBM has demonstrated good feasibility and safety, with only a small fraction (2.1%) of patients experiencing grade 3 or 4 adverse events attributable to the vaccine.^105^ Additionally, certain subsets of patients may derive long‐term benefits from DCVax‐L, particularly those harboring a methylated MGMT promoter, who exhibited a prolonged median overall survival of 34.7 months and a 3‐year survival rate of 46.4%.^106^ DC vaccine therapy has also been observed to elicit systemic and intracranial T‐cell responses influenced by the local CNS tumor microenvironment (TME), showing particular efficacy in patients lacking bulky, actively progressing tumors and displaying low TGF‐β2 expression levels. Furthermore, it has demonstrated safety profiles without dose‐limiting toxicity or serious adverse effects, underscoring its tolerability.^106^ These collective findings represent a pivotal advancement in GBM treatment, offering promise for enhanced survival outcomes through the integration of personalized immunotherapy into the standard care framework.^74^

The α‐type‐1 DC vaccine, composed of DCs loaded with a mixture of synthetic peptides, has demonstrated favorable outcomes in individuals diagnosed with newly onset HGG.^107^ Following vaccine administration, there was an observed increase in the production of interleukin‐12 (IL‐12) by activated DCs, accompanied by positive cytotoxic T lymphocyte (CTL) responses in 67% of patients. Moreover, the treatment exhibited significant survival‐prolonging effects, with 33% of patients surviving beyond 6 years of follow‐up and 13% of them remaining relapse‐free.^107^ These findings suggest that peptide‐cocktail‐pulsed α‐type‐1 DC vaccines hold potential therapeutic efficacy in the management of glioma patients.

Patients with recurrent malignant gliomas can also benefit from DC vaccines. The Wilms' tumor 1 (WT1)‐pulsed DC vaccination therapy derived stable disease in 50% of the patients treated with WT1‐pulsed DC vaccination, with neurological improvements and tumor shrinkage observed in 20% of these patients.^108^ Additionally, the therapy was well‐tolerated with no serious adverse events reported, and immunological analysis detected WT1‐reactive cytotoxic T cells in patients treated with WT1‐pulsed therapy, indicating an immune response.^108^ Positivity for skin reactions at injection sites remained high throughout the treatment course, demonstrating the feasibility and safety of WT1‐pulsed DC vaccination therapy in managing relapsed malignant gliomas. These findings warrant further investigation into the safety and efficacy of DC vaccines in larger‐scale clinical trials.

Patients diagnosed with malignant glioma may undergo treatment involving DC vaccines. Research indicates that autologous dendritic cell‐tumor vaccine therapy has shown promising outcomes, including initial tumor shrinkage, elevated levels of tumor‐infiltrating CD8(+) lymphocytes, and improved median survival rates (525 days) and 5‐year survival rates (18.8%) in patients with grade IV glioma compared to historical control cohorts (median survival of 380 days and 5‐year survival of 0%).^109^ Furthermore, patients with recurrent malignant glioma have also demonstrated benefits from DC vaccines. Wilms' tumor 1 (WT1)‐pulsed DC vaccination therapy resulted in stable disease in 50% of patients, with neurological enhancements and tumor regression observed in 20% of these cases.^108^ The therapy was well‐tolerated, with no severe adverse effects reported, and immunological analysis detected WT1‐reactive cytotoxic T cells in patients treated with WT1‐pulsed therapy, indicating an immune response.^108^ Notably, skin reactions at injection sites remained consistently positive throughout the treatment course, demonstrating the feasibility and safety of WT1‐pulsed DC vaccination therapy in managing relapsed malignant gliomas. These findings underscore the importance of further investigating the safety and efficacy of DC vaccines through larger‐scale clinical trials. The positive outcomes of glioma vaccines have been summarized in Table 2.

### 6.2 Negative outcomes and adverse effects

While previous clinical trials have shown promising results regarding the use of vaccines in glioma treatment, it is essential to address the negative outcomes observed. These may include adverse events and limited survival benefits.

Despite the overall favorable response noted in earlier investigations, it is crucial to acknowledge the occurrence of adverse events, albeit infrequently. For instance, in the context of the IMA950 vaccine administered to patients with GBM, although generally well‐tolerated, some participants experienced minor adverse events such as reactions at the injection site, rash, and fatigue.^102^ Additionally, isolated cases of more severe adverse events like allergic reactions, anemia, and anaphylaxis were reported. Notably, two patients encountered grade 3 dose‐limiting toxicities, specifically fatigue, and anaphylaxis.^102^ It is imperative to recognize the potential occurrence of these adverse events and take appropriate measures to mitigate them, ensuring the safety and tolerability of vaccine‐based therapies for glioma patients.

Moreover, although the autologous DC tumor vaccine therapy described exhibits promise in the treatment of malignant glioma, adverse outcomes have been observed in the clinical trial.^109^ Primarily, there was a transient increase in aspartate aminotransferase (AST) and alanine aminotransferase (ALT) levels in approximately 47.1% of treated patients. While this elevation was reversible, it hints at potential hepatotoxicity linked to the therapy.^109^ These adverse outcomes underscore the presence of challenges and limitations in the efficacy and safety of glioma vaccine therapy, necessitating further investigation and clinical trials to address these concerns.

Furthermore, despite observed enhancements in median survival and 5‐year survival rates compared to historical control groups, the OS benefits of DC tumor vaccine therapy in treating malignant glioma remain limited.^109^ For instance, the 5‐year survival rates for patients with grade IV glioma was 18.8%, suggesting that a considerable proportion of patients succumbed to the disease eventually.^109^ This trend persisted in the application of autologous dendritic cell‐based immunotherapy for newly diagnosed GBM patients.^110^ Although the clinical trial demonstrated the feasibility of integrating the vaccine into standard care treatment without major toxicities, the outcomes in terms of PFS and OS, while improved compared to some historical controls, fell short of the transformative impact anticipated at the trial's commencement.^110^ This finding serves as a poignant reminder of GBM's resistance to current therapeutic modalities and emphasizes the pressing need for treatments capable of more effectively overcoming the tumor's defenses.

Similarly, the use of personalized vaccine therapy, which harnesses the patient's own tumor antigens to elicit an immune response, yielded comparable outcomes.^111^ Although the AFTV was well‐tolerated and exhibited a favorable safety profile, with no treatment‐related adverse effects surpassing Grade 1 severity, the absence of severe toxicity must be juxtaposed against the overall modest impact on disease progression and survival. This comparison prompts crucial inquiries regarding the delicate balance between treatment tolerability and clinical effectiveness in the context of GBM, where the urgent demand for more efficacious therapies often pushes the boundaries of acceptable risk.^112^

The introduction of peptide‐based vaccines for children with recurrent HGGs sheds light on the additional complexities surrounding vaccine therapy in glioma treatment.^112^ Despite demonstrating safety and the ability to stimulate tumor‐specific immune responses, clinical outcomes underscore the challenging nature of treating pediatric gliomas. The median PFS of 4.1 months and OS of 12.9 months elucidate the limited efficacy of the vaccine in altering the disease trajectory.^112^ Furthermore, the occurrence of symptomatic pseudoprogression in one patient underscores the challenges of assessing vaccine efficacy using conventional imaging techniques, which may misinterpret treatment‐induced inflammatory responses as tumor progression.^112^

Across these trials, several critical themes emerge. Firstly, the modest enhancements in survival metrics highlight the imperative for more potent and precisely targeted vaccine formulations capable of eliciting stronger and more enduring immune responses. Next, the trials underscore the necessity of devising improved methodologies for monitoring treatment responses, extending beyond conventional imaging techniques, to accurately capture the biological ramifications of vaccine therapies on the tumor. Additionally, while adverse effects are generally mild, they underscore the significance of closely monitoring patient safety, particularly as vaccine therapies are frequently administered concurrently with or subsequent to standard treatments that possess their own toxicity profiles. Comprehending and mitigating these adverse effects is paramount to ensuring that the potential advantages of vaccine therapies are not overshadowed by their associated risks. The negative outcomes and adverse effects following glioma vaccines have been illustrated in Table 3.

**TABLE 3:** Positive and negative outcomes of glioma vaccines.

Outcome	Description
**Positive outcomes**	
Favorable Survival Rates and Prolonged Overall Survival^48^, ^102^, ^103^, ^109^	IMA950 and SurVaxM vaccines demonstrated PFS of 74% and 95.2% at 6 months in newly diagnosed GBM patients respectively SurVaxM vaccine showcased an average PFS of 11.4 months SurVaxM, DCVax‐L, and ERC1671 vaccines demonstrated a median OS of 25.9, 23.1, and 12 months, respectively Autologous dendritic cell‐tumor vaccine therapy resulted in improved median survival rates (525 days) and 5‐year survival rates (18.8%) in grade IV glioma patients
Restricted Incidence of Adverse Events^48^, ^49^	Peptide‐based vaccines, including SurVaxM and IDH1‐vac, demonstrated limited adverse events, primarily grade 1, underscoring their safety and efficacy
Enhanced Immunogenicity and Immune Responses^49^, ^50^, ^102^	The IMA950 vaccine activated the immune system robustly, with sustained responses of central memory CD8+ T cells The IDH1‐vac showed immune responses in 93.3% of patients with IDH1(R132H) + astrocytomas GAPVAC DNA/RNA‐based vaccines induced sustained responses of central memory CD8+ T cells and predominantly CD4+ T cell responses against predicted neoepitopes, leading to enhanced immunogenicity
**Negative outcomes**	
Limited Survival Benefits^109^, ^112^	5‐year survival rates remain limited – 18.8% in grade IV glioma patients Autologous dendritic cell‐based immunotherapy for newly diagnosed GBM patients fell short of the transformative impact anticipated, with modest improvements in PFS and OS Peptide‐based vaccines for children with recurrent high‐grade gliomas showed limited efficacy, with median PFS of 4.1 months and OS of 12.9 months
Presence of Adverse Events^102^, ^109^	IMA950 vaccine induced minor adverse events such as reactions at the injection site, rash, and fatigue Isolated cases of more severe adverse events like allergic reactions, anemia, and anaphylaxis were reported Autologous DC tumor vaccine therapy resulted in a transient increase in AST and ALT levels in 47.1% of treated patients, hinting at potential hepatotoxicity linked to the therapy

Abbreviations: ALT, alanine aminotransferase; AST, aspartate aminotransferase; OS, overall survival; PFS, progression‐free survival.

## 7 CHALLENGES WITH VACCINE IMPLEMENTATION

### 7.1 Therapeutic resistance: heterogeneity and immune evasion mechanisms

The development and effectiveness of vaccines for gliomas, notably GBM, are hindered by therapeutic resistance stemming from tumor heterogeneity and immune evasion mechanisms. GBM's complexity arises from its diverse microenvironment, which fosters cellular phenotypes and genetic variances that contribute to resistance to therapies. These tumors evade immune detection through strategies like PD‐L1 upregulation and immunosuppressive cytokine secretion, undermining the immune system's ability to target and eliminate tumor cells.^113^, ^114^, ^115^ Glioma vaccines aim to prime the immune system against tumor‐specific antigens, yet their efficacy is compromised by the tumor's ability to suppress immune responses and the inherent heterogeneity that allows some cells to escape antigen‐targeted immunity.^116^, ^117^ This complexity underscores the need for innovative strategies to overcome the barriers to effective glioma vaccine development and implementation.

### 7.2 Absence of standardized guidelines

The lack of standardized guidelines for evaluating glioma vaccine responses complicates neuro‐oncology, especially due to gliomas' inherent heterogeneity. The challenge is exacerbated by the immunosuppressive microenvironment characteristic of GBMs, which features extensive heterogeneity and systemic immunosuppression. This diversity, alongside varying vaccine formulations, makes standardizing efficacy assessments difficult, further obscured by the absence of agreed‐upon immunological endpoints to gauge clinical benefit.^22^, ^23^, ^118^ Moreover, the dynamic and variable nature of the immune response to glioma vaccines, which can be affected by prior treatments and the patient's overall immune health, demands a nuanced and comprehensive methodology for evaluating vaccine success.^119^, ^120^

### 7.3 Ethical and social considerations: legal and regulatory issues and hesitancy in acceptance from patients

Glioma vaccine development introduces ethical considerations.^49^ As with all vaccine deployment, at its core is obtaining informed consent from patients.^121^ The experimental nature of glioma vaccine approaches necessitates understanding the potential risks and benefits, ensuring that patients are well‐informed participants in the trials.^122^ Achieving a balance between advancing management and safeguarding patient autonomy is a challenge that must be overcome for successful implementation. The reluctance or refusal to receive vaccines, a phenomenon observed notably during the COVID‐19 pandemic, could also impact the acceptance of glioma vaccines.^123^ Similarly, equitable access to glioma vaccine trials is another challenge. Most available studies have been conducted in HICs.^74^ This raises questions about the generalizability of the findings, the inclusivity of diverse patient populations, and the suitability of such vaccines in low‐resource settings.

Furthermore, the social considerations surrounding gliomas are marked by stigma. The stigma associated with glioma and its treatments creates barriers to acceptance, influencing how patients approach participation in vaccine trials.^124^ The intersection of cultural beliefs and societal norms influences patient acceptance and participation in vaccine trials.^123^ The adaptive nature of glioma vaccines, often tailored to individual patient profiles, introduces challenges to traditional regulatory frameworks.^49^ Adapting these frameworks to accommodate the unique characteristics of personalized cancer vaccines is a challenge. Moreover, regulatory approval processes vary globally, leading to challenges in harmonizing standards for glioma vaccines.^125^ Achieving consistency in regulatory requirements across different jurisdictions is a complex task that impacts the timeline and feasibility of global clinical trials and acceptance.^125^ The challenges in glioma vaccine implementation have been summarized in Table 4.

**TABLE 4:** Challenges in glioma vaccine implementation.

Challenge	Description
Therapeutic Resistance: Heterogeneity and Immune Evasion^113^, ^114^, ^115^, ^116^	Glioma vaccine development is hindered by tumor heterogeneity and immune evasion mechanisms, including upregulation of PD‐L1, secretion of immunosuppressive cytokines, and recruitment of regulatory T cells. This complexity necessitates innovative strategies to overcome barriers to vaccine efficacy
Absence of Standardized Guidelines^118^, ^119^, ^120^	The lack of standardized guidelines for assessing vaccine responses complicates the evaluation of vaccine efficacy, especially given gliomas' heterogeneity and the immunosuppressive tumor microenvironment. The diversity of vaccine formulations adds to the challenge of creating universal assessment criteria
Ethical and Social Considerations: Legal, Regulatory Issues, and Patient Hesitancy^121^, ^122^, ^123^, ^124^, ^125^	Ethical and social considerations include obtaining informed consent, addressing vaccine hesitancy, and ensuring equitable access to vaccine trials. The personalized nature of glioma vaccines and the variability in regulatory approval processes pose additional challenges

Abbreviation: PD‐L1, programmed death‐ligand 1.

## 8 DISCUSSION AND FUTURE PROSPECTS

In order to tackle the challenges surrounding glioma vaccines and unlock their full potential in glioma treatment, promising strategies such as combination therapies and targeted approaches like personalized and mRNA vaccines are being explored. Additionally, ensuring ethical considerations and equitable access through harmonized regulatory standards and equitable participation is crucial for advancing glioma therapy responsibly and maximizing its impact worldwide.

### 8.1 Combination therapy

Combination therapies that pair vaccines with radiotherapy or chemotherapy offer a promising strategy for enhancing glioma treatment. These therapies aim to amplify the therapeutic efficacy of the standard care regimen—surgical resection followed by radiotherapy and chemotherapy—by activating the immune system to target and eliminate tumor cells. Radiotherapy, combined with vaccines, has shown potential for upregulating tumor antigen expression and enhancing immune cell infiltration into the TME. This synergy improves tumor immunogenicity and vaccine efficacy, potentially leading to better tumor control and extended survival for patients.^126^ Similarly, administering low‐dose chemotherapeutic agents before vaccination can modulate the immune response, reducing immunosuppressive cells and enhancing tumor antigen presentation. This approach strengthens the vaccine‐induced anti‐tumor immune response.^127^ However, clinical trials investigating these combination therapies have yielded mixed outcomes, showcasing enhanced survival rates in select cases while underscoring challenges such as overcoming the immunosuppressive milieu of GBM and navigating the intricate TME.^22^ Ongoing research endeavors aim to optimize the timing, dosage, and sequence of these therapies to maximize their therapeutic potential for glioma patients.

### 8.2 Targeted therapy

Progress in glioma vaccine research is uncovering promising candidates and immune biomarkers that have the potential to significantly enhance treatment outcomes. Clinical trials investigating personalized vaccines tailored to target both unmutated antigens and neoepitopes have demonstrated robust immune responses.^50^ These vaccines play a critical role in activating T cells and attenuating immune checkpoint inhibition, thereby fostering heightened anti‐tumor immunity.^50^ Moreover, the identification of glioma antigens such as TP53, IDH1, C3, and TCF12 has been instrumental in the development of mRNA vaccines capable of eliciting effective immune responses against gliomas.^128^ Personalized neoantigen vaccines have been shown to induce the production of circulating polyfunctional neoantigen‐specific CD4+ and CD8+ T cell responses, characterized by a memory phenotype, and enhance T cell infiltration into the tumor microenvironment.^51^

Furthermore, collaborative initiatives such as the Accelerating COVID‐19 Therapeutic Interventions and Vaccines (ACTIV) partnership underscore the value of coordinated efforts among biotechnology and pharmaceutical industries, governmental agencies, and academia. Through harmonized, randomized controlled trials, this partnership seeks to expedite vaccine development and distribution, providing a potential model for future glioma vaccine research.^129^ These advancements underscore the multifaceted potential of glioma vaccines in facilitating immune cell infiltration into the tumor microenvironment, offering promising avenues for further exploration and development.

### 8.3 Personalized medicine

The emergence of highly specific glioma vaccines, such as the SurVaxM peptide vaccine and the IDH1(R132H)‐specific vaccine, presents an opportunity to advance personalized medicine.^48^, ^49^ Next‐generation sequencing (NGS) technologies, including whole exome sequencing (WES) and whole genome sequencing (WGS), offer valuable tools for identifying predictive biomarkers associated with glioma vaccine response and prognosis.^130^ Additionally, artificial intelligence (AI) and machine learning (ML) hold promise for revolutionizing glioma vaccine development by enabling the prediction of tumor grading and genomics from imaging data, automating histopathological diagnosis, and offering insights into prognosis.^131^ Leveraging these innovative technologies facilitates the administration of precise glioma vaccines. Hence, ongoing efforts should focus on identifying immune biomarkers, genetic signatures, and imaging biomarkers to guide patient selection, monitor treatment response, and predict long‐term outcomes.

### 8.4 Ethical considerations and equitable distribution

Addressing the ethical and social challenges inherent in glioma vaccine research and application necessitates a global commitment to harmonizing regulatory standards and ensuring equitable participation.^73^, ^132^ This entails fostering transparency for consent, safeguarding privacy, promoting inclusivity, and ensuring adaptability in clinical trials and regulatory practices to ensure that glioma vaccine development progresses in an ethically and socially responsible manner. By adhering to ethical frameworks and guidelines, researchers can ensure that glioma vaccine research is conducted responsibly, respecting patient autonomy, and dignity while advancing scientific progress.

Emphasizing global collaboration and access is crucial, particularly in low‐ and middle‐income countries (LMICs) where access to optimal glioma treatments may be limited. Findings from LMICs suggest that gliomas may present distinctively, potentially leading to different prognoses and survival outcomes depending on mutation status and the extent of resection.^133^ This underscores the importance of conducting research on advanced glioma treatments, such as glioma vaccines, in LMICs, as the population phenotype may differ from that in high‐income countries (HICs). Initiatives aimed at improving glioma vaccine access, affordability, and distribution on a global scale are imperative to address disparities in healthcare access and ensure equitable outcomes for glioma treatment. The Global Vaccine Action Plan has identified the WHO Global Vaccine Safety Blueprint as its vaccine safety strategy.^134^ Synergies and resource mobilization opportunities presented by the Decade of Vaccines can enhance monitoring and response to vaccine safety issues, thereby leading to more equitable delivery of vaccines worldwide.

Patient‐centered care is imperative in glioma vaccine development and implementation. Clinicians must consider both tumor characteristics and patients' personal criteria for a holistic treatment plan.^135^ It is suggested that official recommendations should only serve as a guide, and tumor boards should provide consultative proposals without becoming too oppressive, particularly concerning medico‐legal issues.^135^ This is essential to encourage innovation and imperative for the development of novel treatments such as glioma vaccines for a disease that cannot yet be cured. Therefore, patient education and empowerment become imperatives throughout their treatment journey. The future prospects of glioma vaccines have been summarized in Table 5.

**TABLE 5:** Future prospects in the development of glioma vaccines.

Future prospect	Description
Combination Therapy^23^, ^126^, ^127^	Combination therapies involving vaccines with radiotherapy or chemotherapy offer a promising strategy for enhancing glioma treatment Radiotherapy combined with vaccines may upregulate tumor antigen expression and enhance immune cell infiltration into TME Administering low‐dose chemotherapeutic agents before vaccination can modulate the immune response, strengthening the vaccine‐induced anti‐tumor immune response
Targeted Therapy^50^, ^51^, ^128^, ^129^	Personalized vaccines targeting unmutated antigens and neoepitopes have demonstrated robust immune responses, activating T cells and mitigating immune checkpoint inhibition Identification of glioma antigens like TP53, IDH1, C3, and TCF12 has been instrumental in developing mRNA vaccines capable of eliciting effective immune responses against gliomas Personalized neoantigen vaccines induce the generation of circulating polyfunctional neoantigen‐specific CD4+ and CD8+ T cell responses, enhancing T cell infiltration into the TME Collaborative initiatives like the ACTIV partnership aim to expedite vaccine development and distribution, offering a model for future glioma vaccine research
Personalized Medicine^48^, ^49^, ^130^, ^131^	Highly specific glioma vaccines, such as the SurVaxM peptide vaccine and IDH1(R132H)‐specific vaccine, present an opportunity for personalized medicine NGS technologies like WES and WGS aid in identifying predictive biomarkers associated with glioma vaccine response and prognosis AI and ML hold promise for revolutionizing glioma vaccine development by predicting tumor grading and genomics, automating diagnosis, and providing prognosis insights
Ethical Considerations and Equitable Distribution^132^, ^133^, ^134^, ^135^	Addressing ethical and social challenges in glioma vaccine research requires a global commitment to harmonizing regulatory standards and ensuring equitable participation Global collaboration and access are crucial, especially in LMICs, to address disparities in healthcare access and ensure equitable outcomes for glioma treatment Patient‐centered care, innovation encouragement, and patient education are imperative throughout the glioma vaccine development and implementation process

Abbreviations: AI, artificial intelligence; ML, machine learning; NGS, next‐generation sequencing; TME, tumor microenvironment; WES, whole exome sequencing; WGS, whole genome sequencing.

## 9 CONCLUSION

Management of gliomas, particularly GBM, remains a significant challenge due to their aggressive nature and resistance to current therapies. Glioma vaccines have emerged as a promising therapeutic strategy, aiming to stimulate the immune system to recognize and eliminate glioma cells. Clinical trials have shown that glioma vaccines can elicit immune responses against tumor cells, suggesting potential benefits as an adjunct to standard treatments. However, challenges such as tumor heterogeneity, immune evasion by gliomas, and the complexity of effectively delivering vaccines remain significant hurdles. Future research must focus on overcoming these barriers, optimizing vaccine formulations, and integrating vaccines into multimodal treatment strategies. Advances in genetic engineering, immunotherapy, and precision medicine hold promise for enhancing the efficacy of glioma vaccines. Continued collaboration across research disciplines is essential for translating these advances into clinically effective treatments for glioma patients.

## AUTHOR CONTRIBUTIONS

WAA, MHS, JKT. Material preparation, data collection, analysis, and writing of the first draft: WAA, MHS, JKT, SR, VS, KD, POT, BBA, AA, VS, NA, MK, TA, OA. Writing and approval of the final draft of the manuscript: All authors.

## FUNDING INFORMATION

The authors declare that no funds, grants, or other support were received during the preparation of this manuscript.

## CONFLICT OF INTEREST STATEMENT

The authors have no relevant financial or non‐financial interests to disclose.

## Data Availability

Data sharing is not applicable to this article as no new data were created or analyzed in this study.
